# Recombination Rate Variation and Infrequent Sex Influence Genetic Diversity in *Chlamydomonas reinhardtii*

**DOI:** 10.1093/gbe/evaa057

**Published:** 2020-03-17

**Authors:** Ahmed R Hasan, Rob W Ness

**Affiliations:** e1 Department of Cell and Systems Biology, University of Toronto, Ontario, Canada; e2 Department of Biology, University of Toronto Mississauga, Ontario, Canada

**Keywords:** recombination rate variation, selection at linked sites, frequency of sex, *Chlamydomonas*

## Abstract

Recombination confers a major evolutionary advantage by breaking up linkage disequilibrium between harmful and beneficial mutations, thereby facilitating selection. However, in species that are only periodically sexual, such as many microbial eukaryotes, the realized rate of recombination is also affected by the frequency of sex, meaning that infrequent sex can increase the effects of selection at linked sites despite high recombination rates. Despite this, the rate of sex of most facultatively sexual species is unknown. Here, we use genomewide patterns of linkage disequilibrium to infer fine-scale recombination rate variation in the genome of the facultatively sexual green alga *Chlamydomonas reinhardtii*. We observe recombination rate variation of up to two orders of magnitude and find evidence of recombination hotspots across the genome. Recombination rate is highest flanking genes, consistent with trends observed in other nonmammalian organisms, though intergenic recombination rates vary by intergenic tract length. We also find a positive relationship between nucleotide diversity and physical recombination rate, suggesting a widespread influence of selection at linked sites in the genome. Finally, we use estimates of the effective rate of recombination to calculate the rate of sex that occurs in natural populations, estimating a sexual cycle roughly every 840 generations. We argue that the relatively infrequent rate of sex and large effective population size creates a population genetic environment that increases the influence of selection on linked sites across the genome.

## Introduction

Recombination is both a fundamental evolutionary process and required to ensure proper disjunction of chromosomes during meiosis. Meiotic recombination has two possible outcomes: crossing over (CO) and noncrossing over, also known as gene conversion. At the population level, recombination reduces interference between linked adaptive and harmful mutations and is therefore an important determinant of how well natural selection can act (Hill and Robertson 1966). There is clear evidence that recombination rate varies at multiple scales across nature, with variability observed within and between taxa (de Massy 2013; Stapley et al. 2017) as well as within the genome (Nachman 2002; Choi and Henderson 2015). Across the genome, COs often cluster in localized elevations known as “hotspots” (Choi and Henderson 2015). This fine-scale variation means that local recombination rate may substantially affect the rate of adaptation and accumulation of harmful mutations at a locus (Felsenstein 1974; McVean et al. 2004).

A major determinant of recombination rate in a population is the frequency with which sex occurs. Even if physical recombination rate is high, the effective rate of recombination in a population may be lower due to infrequent sex or high rates of inbreeding (Charlesworth and Wright 2001; Wright et al. 2008). Reduced opportunity for recombination between heterozygous chromosomes should extend linkage over larger tracts of the genome than would be expected for species with large effective population sizes and high physical recombination rates. In species that switch between asexual and sexual generations, as is the case in many eukaryotic microbes (Dacks and Roger 1999; Weedall and Hall 2015), the influence of selection at linked sites in determining patterns of diversity will vary with not only the physical rate of recombination but also the frequency of sex in the population (Hartfield 2016). The influence of selection at linked sites may explain why genetic diversity is constrained to a narrow range in nature relative to the scale of variation in population size (Maynard Smith and Haigh 1974; Coop and Ralph 2012; Corbett-Detig et al. 2015; but see Coop 2016), especially when considering protists with extremely large census population sizes (e.g., Filatov 2019).

Despite the fact that protists represent the majority of eukaryotic diversity (Burki et al. 2008), recombination in protists has seen little investigation. A recent review reports only nine linkage maps from species in the Stramenopiles–Alveolates–Rhizaria supergroup, as opposed to over 300 across animals and plants (Stapley et al. 2017). Although laboratory crosses indicate generally high rates of recombination in protists (*∼*10–50 cM/Mb, e.g., Heesch et al. 2010; Blake et al. 2011), these estimated rates do not consider the influence of frequency of sex in nature. It is estimated that the frequency of sex is unknown in the vast majority (>99%) of free-living protist species (Weisse 2008), to the point where it is unknown whether many are sexual at all (Schurko et al. 2009; D’Souza and Michiels 2010). Although very difficult to measure in nature, the rate of sex can be estimated as the relative frequency of meioses to mitoses by combining direct estimates of the recombination (*r*) and mutation (*μ*) rate with population estimates of genetic diversity (4*N*_e_*μ*) and the effective recombination rate ($4N_{e}r$) (Tsai et al. 2008). To date, this technique has only been used in a limited set of organisms (e.g., Tsai et al. 2008; Grimsley et al. 2010) because there are very few in which diversity (*θ*), population recombination rate (*ρ*), physical recombination rate (*r*), and mutation rate (*μ*) are known.

Here, we use population genomic data from the unicellular, facultatively sexual green alga *Chlamydomonas reinhardtii* to examine fine-scale variation in the population recombination rate across the genome. We also integrate laboratory-based estimates of mutation and physical recombination rate to infer the frequency of sex in nature. Specifically, we address the following questions: 1) What is the landscape of recombination rate variation across the genome of *C. reinhardtii*? 2) What genomic features predict recombination rate variation in the genome? 3) What is the rate of sex in natural populations of *C. reinhardtii*? and 4) How does recombination rate affect patterns of neutral diversity?

## Materials and Methods

### Strains, Sequencing, and Alignment

Here we use genome sequence from 19 (9 *MT+*, 10 *MT−*) natural strains of *C. reinhardtii*, sampled from Quebec, Canada. For strains CC-2935, CC-2936, CC-2937, and CC-2938, we obtained publicly available sequencing data (Flowers et al. 2015), whereas the remainder of the strains were originally published in Ness et al. (2016). These 19 haploid strains (supplementary table S1, Supplementary Material online) are all sampled from two nearby localities in Quebec and show no evidence of population structure (Craig et al. 2019). We aligned 100-bp paired-end reads with the Burrows-Wheeler Aligner 0.7.4-r385 (Li and Durbin 2009); as the *C. reinhardtii* reference genome is derived from an *MT+* individual and contains no organelle genomes, we appended the *MT−* locus and organelle genomes to the reference to allow mapping of reads derived from these regions. The GATK v3.3 tools HaplotypeCaller and GenotypeGVCFs were then used to call single nucleotide polymorphisms (SNPs) and short indels and stored in Variant Call Format files (nondefault settings: ploidy = 1, includeNonVariantSites=true, heterozygosity = 0.02, indel_heterozygosity = 0.002). Ploidy was set to 1 because all strains included are haploid. Heterozygosity values were approximately equal to the genomewide average rate of polymorphism ($\theta_{\pi }$) across silent and coding sites in the Quebec subpopulation (Craig et al. 2019).

Of the 6,497,950 SNPs identified, we retained 4,736,814 high-quality SNPs for recombination rate estimation after using the following site filters: 1) mean genotype quality across all calls >30, 2) only one alternate allele identified (i.e., diallelic), and 3) minor allele frequency >0.1 (to exclude singletons). All filtering was performed with bcftools (https://github.com/samtools/bcftools; last accessed April 1st, 2020) and cyvcf2 (Pedersen and Quinlan 2017).

### Estimation of Recombination Rate Variation

To obtain chromosomewide maps of recombination rate variation in the *C. reinhardtii* genome, we used LDhelmet 1.9 (Chan et al. 2012), which calculates fine-scale estimates of population recombination in intervals bounded by adjacent SNPs. The coalescent-based approach of LDhelmet allows for inference of ancestral recombination rate variation. LDhelmet reports the population recombination rate $\rho =2N_{e}r$ which reflects the size of the recombining population (*N*_e_) and the physical recombination rate in sexual generations (*r*, recombination events bp^−1^ generation^−1^).

### Mutation Matrix Estimation

LDhelmet incorporates a quadra-allelic mutation model, allowing for transition probabilities between the four nucleotides to be specified and taken into account in recombination rate inferences. The method for estimating this transition matrix specified by Chan et al. (2012) involves comparison with an outgroup to designate ancestral alleles. However, because no outgroup genome is currently available for *C. reinhardtii*, we instead used data from a prior mutation accumulation study in *C. reinhardtii* (Ness et al. 2015) to estimate transition probabilities. Using a data set of 5,710 single nucleotide mutations, we designated the prior states of mutated sites within each strain as “ancestral” and otherwise followed the method of Chan et al. (2012) to estimate the mutation matrix, which was as follows (with both rows and columns ordered as A, C, G, T):

$$\left[\begin{array}{cccc} 0.61 & 0.11 & 0.19 & 0.09\\ 0.26 & 0 & 0.22 & 0.52\\ 0.5 & 0.22 & 0.03 & 0.25\\ 0.11 & 0.2 & 0.11 & 0.57 \end{array}\right].$$

### Block Penalty

The block penalty parameter in LDhelmet determines the level of smoothing of *ρ* estimates along the chromosomes. To ascertain the optimal block penalty for our data set, we adapted a Python script from Singhal et al. (2015) to perform simulations with the coalescent-based simulator macs-0.5d (Chen et al. 2009). We simulated haplotypes under a range of six background recombination rates (*ρ *= 0.0001, 0.001, 0.01, 0.1, 1.0, and 2.5). These values were based on the range of values observed in preliminary LDhelmet runs with default parameters. For each of these background recombination rates, we simulated 19 1-Mb sequences, assigning bases to derived alleles using the mutation matrix estimated above. Additionally, following Singhal et al. (2015), we placed eight 2-kb recombination hotspots along the length of each sequence, with two hotspots each of relative fold increases 10×, 20×, 40×, and 60×. We used a window size of 2 kb since hotspots are 2 kb or larger in many species (Kim et al. 2007; Coop et al. 2008; Singhal et al. 2015; Booker et al. 2017). To test for the effect of variation in hotspot length, or proportion of hotspots in the genome, we repeated these simulations with 4 and 6 kb hotspot sizes. Each simulation was repeated ten times. We then ran LDhelmet across block penalties 5, 10, 50, and 100 on all simulated haplotypes and compared the observed LDhelmet recombination estimates with expected recombination rates. For our LDhelmet runs, we used a *θ* value of 0.03, based on a prior empirical estimates of neutral diversity (Flowers et al. 2015; Craig et al. 2019). Comparing observed *ρ* with our known recombination rates, we found that a block penalty of 100 yielded a ratio of observed/expected *ρ* closest to 1 across all simulated recombination rates (supplementary fig. S1, Supplementary Material online).

### Power to Detect Hotspots

To detect recombination hotspots from LDhelmet *ρ* estimates, we modified the “find_hotspots.py” Python script from Singhal et al. (2015) that summarizes *ρ* values in nonoverlapping windows. Hotspots are elevations in recombination rate at a locus compared with its surrounding regions; however, whether or not a region is classified as a hotspot will be affected by both the block penalty as well as the size of the flanking regions. To determine the optimal block penalty and flank size to best detect recombination hotspots in our data, we averaged the LDhelmet outputs from our simulations above into nonoverlapping 2-kb windows. For each window, we also calculated recombination rates on either flank over various flank sizes (ranging from 20 to 100 kb in increments of 20 kb). Then, we examined how many of our known simulated hotspots across all combinations of block penalty values and flank sizes demonstrated at least a 5-fold increase in *ρ* compared with the flanks. We found that a block penalty of 10 and a flank size of 100 kb provided the highest power (1 − number of false negatives) to detect hotspots across all simulated background *ρ* values, including ρ=0.001, which is the closest to expected recombination rate in *C. reinhardtii* following a preliminary LDhelmet run (supplementary fig. S2, Supplementary Material online). Based on these simulations, our power to detect hotspots is relatively high (*∼*0.9). Following these simulations, we defined hotspots as regions that 1) are at least 2 kb in length and 2) exhibit a mean 5-fold increase in *ρ* compared with the surrounding 200 kb of sequence, similar to previous approaches (International HapMap Consortium 2005; Singhal et al. 2015; Booker et al. 2017).

### Estimating the Recombination Landscape

To estimate the genomic landscape of recombination, we ran LDhelmet twice for 1,000,000 iterations on each chromosome of *C. reinhardtii* following 100,000 iterations of burn-in. The two runs were performed with the same parameters save for the block penalty. In the first run, which was used to estimate the landscape of genomewide *ρ*, we used a block penalty of 100. For the second run, we changed the block penalty to ten for better hotspot detection given the results of our simulations (see above). As with our simulations, we used a *θ* value of 0.03 (Craig et al. 2019) and the mutation matrix shown above in both runs. To detect hotspots, we used our adaptation of the find_hotspots.py Python script (see Data Availability) from Singhal et al. (2015) to summarize LDhelmet output into nonoverlapping 2-kb windows, while also summarizing *ρ* over the surrounding 200 kb for each window. We used the LDhelmet run with a block penalty of 100 for all analyses except for hotspot detection.

### LD Decay across Chromosomes

Pairwise calculations of linkage disequilibrium (*r*^2^) between SNPs within each of *C. reinhardtii*’s 17 chromosomes were conducted using plink 1.90 (Chang et al. 2015). For all pairs of SNPs, plink calculates LD statistics with a maximum likelihood approach described in Gaunt et al. (2007). By default, plink filters out pairs of SNPs with an *r*^2^ value below 0.2; we disabled this filtering. We calculated *r*^2^ for all pairs of SNPs within 100 kb of one another, and modeled the expected decay of LD with distance for each chromosome with nonlinear least squares regression in R (R Core Team, 2017) using the following equation from Appendix 2 of Hill and Weir (1988):

(1) $$E(r^{2})=\frac{10+\Gamma }{22+13\Gamma +\Gamma^{2}}\times [1+\frac{\left(3+\Gamma )(12+12\Gamma +\Gamma^{2}\right)}{n(22+13\Gamma +\Gamma^{2})}],$$

where Γ is the product of population recombination rate *ρ* and distance between sites *d*.

### Genomic Correlates of Recombination Rate

We subclassified genic sites in the reference genome of *C. reinhardtii* as protein-coding sequence (CDS), introns, and UTRs. Intergenic sites were subclassified as being within 2 kb upstream of a gene (“flank”, i.e., gene proximate) or more than 2 kb from the nearest gene (“nonflank”). Upon finding that *ρ* in sites upstream of a gene varied based on intergenic tract length (see Results), we binned intergenic tracts by size into tracts <2 kb (i.e., flanked by genes within 2 kb on either side) and tracts >2 kb. For each of the above, average *ρ* was calculated from every corresponding site in the genome. Recombination rates for each annotation (and each bin, in the case of intergenic sequence) were bootstrapped for 1,000 replicates to obtain 95% confidence intervals. For the correlation of GC content with recombination rate, we used a custom Python script to compute GC content in nonoverlapping 2-kb windows.

LD-based estimates of *ρ* are expected to be correlated with SNP density due to the action of background selection and selective sweeps. Although we know that the resolution of the recombination landscape is affected by SNP density, *ρ* estimates from methods such as LDhelmet should be unbiased with respect to SNP density. However, subsampling SNPs has been shown to sometimes cause reductions in *ρ* estimates (Chan et al. 2012). Thus, to ascertain whether there were differences in recombination rate between annotations while controlling for variation in SNP density, we split the genome into tracts of annotations of interest and calculated *ρ* and SNP density for each tract. We then fit a multiple regression model with predictors annotation, SNP density, and their interaction, whereas *ρ* was set as the response variable.

### Recombination and Nucleotide Diversity

Although a correlation between recombination rate and neutral diversity is suggestive of the effects of selection at linked sites, LD-based estimates of *ρ* are inappropriate for correlations with nucleotide diversity because both statistics are scaled by *N*_e_. This means that the two measures will be autocorrelated due to demography, selection, and variance in coalescence times across the genome. We therefore tested the relationship between recombination and diversity using crossover data from Liu et al. (2018), who sequenced 108 offspring from 27 tetrads. First, we examined the concordance between our *ρ* estimates and these COs by testing for an enrichment of COs in genomic regions with high *ρ*. We binned our *ρ* estimates for genomic intervals into 50 equal-sized bins between *ρ* values of 0 and 0.06. For each bin, we counted the number of COs found in regions of the genome corresponding to that range of *ρ* values. The count of COs was converted to an estimate of physical recombination rate in cM/Mb by dividing the number of COs in each bin by the number of individuals in the data set (108), multiplying by 100 (since 1 cM represents a 1% chance of a CO), and then dividing the resulting per individual measure by the number of sites falling within each *ρ* bin.

Next, to examine the relationship between diversity and CO density, we calculated $\theta_{\pi }$ across the genome in 10-kb windows, and then binned these values across 50 bins ranging from $\theta_{\pi }$ values of 0 to 0.1. Windows with <500 silent sites were discarded to reduce noise in diversity estimates, whereas bins with <200 kb were discarded because with so few COs in the data set these bins tended to have zero COs. Then, we assigned COs to bins based on local diversity in the region bounded by each CO and calculated CO rates in cM/Mb as above.

## Results

### The *C. reinhardtii* Recombination Landscape Is Variable and Hotspot-Punctuated

**Fig. 1.**
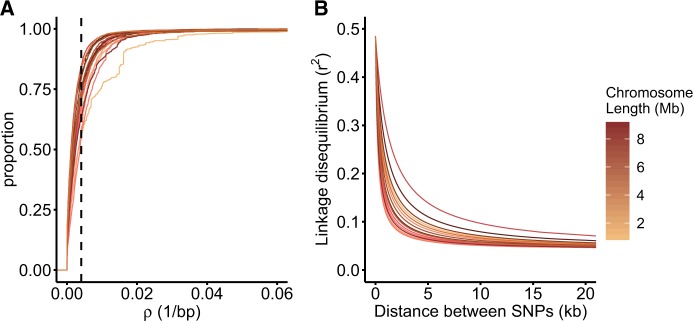
—(*A*) Cumulative frequency distribution of population recombination rate (*ρ*) for each chromosome of *Chlamydomonas reinhardtii*. Each curve represents one of the 17 chromosomes, shaded by chromosome length. *ρ* values were summarized in 2-kb windows. The vertical dashed line indicates the genomewide mean *ρ* value. (*B*) Decay of linkage disequilibrium (*r*^2^) across the 17 chromosomes of *C. reinhardtii*, modeled using the equation provided in Appendix 2 of Hill and Weir (1988).

From our population sample of 19 individuals (supplementary table S1, Supplementary Material online), we calculated fine-scale recombination landscapes across the genome of *C. reinhardtii* with LDhelmet 1.9 (Chan et al. 2012). The genomewide average population recombination rate was *ρ* = 0.0041/bp, and mean recombination rate for each chromosome varied >4.5-fold from 0.0024 to 0.0109, inversely scaling with chromosome lengths (supplementary fig. S3, Supplementary Material online, *R*^2^ = 0.365, *P* = 0.01). *ρ* estimates across the genome were then summarized in nonoverlapping 2-kb windows for fine-scale analysis, with 95% of windows ranging from $\rho =2\times 10^{-5}$ to 0.017; the distribution of recombination rates is shown in figure 1*A*. Analysis of LD decay measured as *r*^2^ dropped to half of their starting value within a mean distance of 577 bp (fig. 1*B*; range 247–1,682 bp). Moreover, the decay of *r*^2^ approached baseline levels within a mean distance of 9,700 bp, where “baseline” was defined as the point at which the instantaneous rate of change in *r*^2^ with physical distance approximates zero to five significant digits (mean *r*^2^ at level off point = 0.074±0.0009).

To examine recombination hotspots, defined as a region that was: 1) >2 kb in length and 2) exhibited a >5-fold increase in *ρ* relative to the flanking 200 kb of sequence. Our hotspot definition was based on a power analysis using simulated sequence data (see Materials and Methods). Under this definition, we found hotspots in all chromosomes, with 875 hotspot regions in total representing 2.75% of the genome, where the average *ρ* within hotspots was more than 26 times the genome average (mean *ρ* at hotspots = 0.1046, mean length = 3,428 bp, mean *ρ* fold increase over local background = 20.6×, mean distance between adjacent hotspots = 122.1 kb).

### Recombination Rates Are Highest Immediately Surrounding Genes

**Fig. 2.**
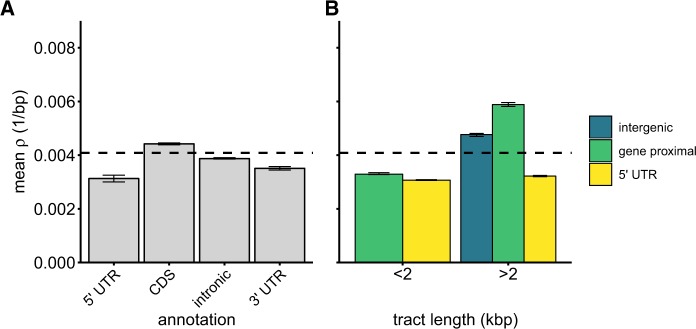
—Population recombination rate (*ρ*) in different annotation categories across the *Chlamydomonas reinhardtii* genome. Error bars represent bootstrapped 95% confidence intervals (*n* = 1,000). The dashed horizontal line represents the mean genomewide *ρ* value. (*A*) Mean *ρ* in genic annotations. (*B*) Mean *ρ* in intergenic sequence and 5′ UTRs by intergenic tract length. The “gene-proximal” annotation represents the 2 kb of sequence at the 3′ end of a given tract (i.e., upstream of the nearest gene), whereas “intergenic” represents the remainder of the tract.

To investigate the correlates of recombination across the genome, we examined how *ρ* varied with different functional annotations in the *C. reinhardtii* reference genome (Merchant et al. 2007). Within genes, we found that *ρ* was significantly higher in coding sequence than introns (fig. 2*A* and supplementary table S2, Supplementary Material online; *β*_coding_ = 0.31, $P=2.2\times 10^{-16}$). UTRs displayed the lowest recombination rates of any annotation (fig. 2*A*, mean *ρ* 5′ UTR = 0.00313, mean *ρ* 3′ UTR = 0.00351). By contrast, we found that intergenic regions had the highest mean *ρ* (= 0.00452) of all annotations.

We found that recombination rate was 12.8% higher in intergenic regions (mean *ρ* = 0.00452) than genic regions (mean *ρ* = 0.00401, Mann–Whitney *U* test, *P* $<2.2\times 10^{-16}$). Within genic DNA, coding exons had significantly higher rates of recombination than introns (Mann–Whitney *U* test, *P* = $3.41\times 10^{-9}$; *ρ* CDS = 0.0044, *ρ* introns = 0.0039).

Within intergenic regions, recombination rate was highest in sequence proximate to genes, with sites within 2 kb of genes displaying 11.7% higher mean *ρ* than the genome background (mean *ρ* of sites within 2 kb of genes = 0.0046). However, we observed that patterns of intergenic recombination varied by intergenic tract length. In the *C. reinhardtii* genome, most intergenic tracts are short, with 50% of all intergenic tracts below 142 bp in size, whereas 91% are below 2 kb (supplementary fig. S4, Supplementary Material online). We observed that in intergenic tracts <2 kb recombination rate was lower than the genomewide average (fig. 2*B*, *ρ* = 0.0036). Conversely, in longer (>2 kb) intergenic tracts, which are less common but represent 9.9% of all genome sequence, recombination rate was slightly higher than the genome average (mean *ρ* = 0.0050). After controlling for SNP density (see above), recombination rate was still significantly higher in longer intergenic tracts than in tracts <2 kb (supplementary table S3, Supplementary Material online; $\beta_{\mathrm{long}}=0.24$, *P* < $2.2\times 10^{-16}$). At the ends of these longer tracts, recombination was highest and nearly 1.5× the genome average (mean *ρ* in 2 kb upstream of genes = 0.0057, mean *ρ* in 2 kb downstream of genes = 0.0061). This trend is also reflected in the UTRs flanking these intergenic tracts: *ρ* in 5′ UTRs adjacent to shorter (<2 kb) intergenic tracts is 4.7% lower than those adjacent to tracts >2 kb.

Intergenic sequence upstream of genes in >4 kb tracts was enriched for hotspots (Fisher’s exact test, odds ratio = 3.25, *P* < $2.2\times 10^{-16}$). 5.6% of all hotspots occur in the 1.9% of the genome corresponding to these sites. We also found a positive correlation between recombination rate and GC content at fine (2 kb) scales (Spearman’s *ρ* = 0.289, *P* $<2.2\times 10^{-16}$) but not at broad (1 Mb) scales (Spearman’s *ρ* = 0.031, *P* = 0.737).

### Recombination Rate Is Correlated with Nucleotide Diversity

**Fig. 3.**
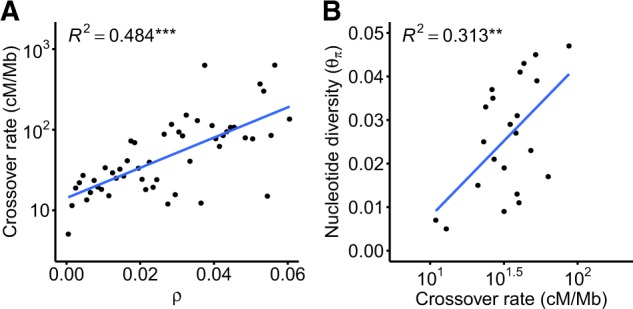
—(*A*) Population recombination rate (*ρ*) is correlated with crossover density, as obtained from the crossover data set of Liu et al. (2018). (*B*) Crossover rates from the Liu et al. (2018) data set correlate with silent site diversity. Nucleotide diversity was calculated at intronic, intergenic, and 4-fold degenerate sites. **P<0.001, ***P<0.0001.

If background selection and selective sweeps are common, we expect to observe reduced neutral diversity in regions of low recombination. Thus, to examine the relationship between recombination and neutral diversity in the *C. reinhardtii* genome, we tested whether COs detected in a prior tetrad sequencing experiment (Liu et al. 2018) tended to colocalize with regions of high *ρ*. Then, we binned our genomewide *ρ* values and assigned COs to each bin to create a measure of physical recombination from CO density (see Materials and Methods) and found a positive and significant relationship between *ρ* and crossover recombination rates (fig. 3*A*, $R^{2}=0.484,P=1.46\times 10^{-8}$), indicating that our LDhelmet *ρ* values reflected real variation in the locations of COs in the genome. Using a similar binning method (see Materials and Methods), we found physical recombination rate and $\theta_{\pi }$ were also significantly correlated (fig. 3*B*, $R^{2}=0.313$, *P* = 0.0068), consistent with widespread reduction of genetic diversity due to the action of selection at linked sites.

### Estimating the Frequency of Sex in *C. reinhardtii*

Due to the fact that mutations can arise each cell division (each meiosis and mitosis) yet recombination only occurs during the fraction of cell divisions that are sexual (*f*), we can use estimates of neutral diversity ($\theta =2N_{e}\mu$) and population recombination rate ($\rho =2N_{e}r$) combined with lab estimates of *μ* and *r* to roughly estimate the relative frequency of meiosis to mitosis, or the frequency of sexual relative to clonal reproduction (Ruderfer et al. 2006; Tsai et al. 2008; Grimsley et al. 2010), as follows:

$$\frac{\text{Number}\;\text{of}\;\text{meioses}}{\text{Number}\;\text{of}\;\text{mitoses}}∼\frac{\rho }{\theta }=\frac{2N_{e}rf}{2N_{e}\mu }.$$

In facultatively sexual species, the realized rate of recombination per generation is the product of physical recombination rate (*r*) and the fraction of generations that are sexual *f* (=meioses/mitoses). We can therefore express the above in terms of *f*:

$$f=\frac{\text{Number}\;\text{of}\;\text{meioses}}{\text{Number}\;\text{of}\;\text{mitoses}}∼\frac{\rho /r}{\theta /\mu }.$$

Thus, our genomewide *ρ* estimate of $4.09\times 10^{-3}$ can be used in tandem with previous estimates of the *C. reinhardtii* recombination rate (*r *=* *12 cM/Mb) (Liu et al. 2018), the mutation rate ($\mu =9.63\times 10^{-10}$) (Ness et al. 2015), and neutral diversity ($\theta =2.75\times 10^{-2}$) (Ness et al. 2016) to solve for *f* and estimate the frequency of sex in natural populations of *C. reinhardtii*. With this approach, we obtain *f *=* *0.001194, corresponding to one sexual generation for every 1/f = *∼*840 asexual generations.

## Discussion

In this study, we have estimated fine-scale recombination rate variation *C. reinhardtii* using patterns of linkage disequilibrium, revealing a recombination landscape punctuated with frequent hotspots. We report a genomewide recombination map for this model species, which offers much higher resolution than a genetic mapping approach would. We found an enrichment of hotspots near genes that leads to an overall increase in recombination rate in intergenic sequence, in concordance with observations in other nonmammalian eukaryotes. Variation in recombination rate across the genome is correlated with nucleotide diversity, suggesting that the influence of selection at linked sites is widespread in the genome and that recombination is a major driver of genetic variation. We have used our estimate of the population recombination rate to estimate the frequency of sex as being once every *∼*840 generations in *C. reinhardtii*, which may in part explain why a species with a relatively high rate of physical recombination and large effective population size experiences such strong effects of selection at linked sites.

Assuming an *N*_e_ of $1.4\times 10^{7}$ (Ness et al. 2016), a genomewide per bp *ρ* of $4.09\times 10^{-3}$ corresponds to an estimate of $r=\rho /2N_{e}=0.015$ cM/Mb. This estimate of genomewide *r* on its own is two orders of magnitude below the estimate of *r* from the genetic map of *C. reinhardtii* (9.15 cM/Mb, Kathir et al. 2003) and most plants (Henderson 2012). The discrepancy between the two estimates of *r* is likely driven by the fact that the frequency of sex is not accounted for in laboratory crosses, since the genetic map measure of *r* is otherwise in line with estimates in other protists (Heesch et al. 2010; Blake et al. 2011; Henderson 2012). Our result shows that in facultatively sexual species, the rate of sex needs to be accounted for in studies of recombination, since laboratory crosses can overestimate the realized *r* (i.e., r′f) in nature.

Between chromosomes, we observe 4.5-fold variation in mean recombination rates, and also find that recombination rate inversely correlates with chromosome length (supplementary fig. S3, Supplementary Material online), a relationship consistent with prior studies in a variety of organisms (Kaback et al. 1992; Kawakami, Smeds, et al. 2014). Given that each chromosome requires at least one crossover event to ensure proper meiotic disjunction (Page and Hawley 2003), it follows that shorter chromosomes exhibit higher per base crossover rates, resulting in more pronounced signatures of LD breakdown, as exemplified in avian recombination (Kawakami, Smeds, et al. 2014). Furthermore, *C. reinhardtii* exhibits moderate rates of LD decay across all 17 chromosomes. Our estimates of the distance (≤10 kb) at which LD (*r*^2^) decays to baseline LD levels are similar to estimates in *Ostreococcus tauri*, another unicellular green alga (*∼*10 kb, Blanc-Mathieu et al. 2017) as well as estimates in *A. thaliana* (*∼*10 kb, Kim et al. 2007), but shorter than in flycatchers (*∼*17 kb, Kawakami, Backström, et al. 2014). We also note that our estimates are shorter than those obtained in a previous study of *C. reinhardtii* that reported a decay to baseline within ∼20 kb (Flowers et al. 2015). The difference between our estimates may be caused by genetic structure in *C. reinhardtii*, where we sequenced isolates all from nearby localities, Flowers et al. utilized a mix of lab strains alongside isolates from a variety of populations across Quebec and Eastern United States. This disparity in our respective estimates is consistent with barriers to recombination across the geographic range of *C. reinhardtii* in North America, with the resulting population structure increasing LD among variants (Craig et al. 2019).

We find numerous recombination hotspots across the genome, similar to observations in mammals, angiosperms, and yeast (Stapley et al. 2017). On the other hand, this recombination profile is unlike that of both *C. elegans*, which has a comparatively homogenous fine-scale recombination landscape (Rockman and Kruglyak 2009; Kaur and Rockman 2014) as well as *D. melanogaster*, which displays some degree of fine-scale heterogeneity but little evidence for highly localized elevations in recombination rate (Chan et al. 2012; Manzano-Winkler et al. 2013). We see elevated recombination and an enrichment of hotspots within regions immediately flanking genes in *C. reinhardtii*, similar to other taxa lacking the PRDM9 protein that determines hotspot locations in humans and mice (Choi and Henderson 2015). Specifically, recombination hotspots upstream of genes have been observed in fungi (Berchowitz et al. 2009; Tsai et al. 2010; Lam and Keeney 2015), finches (Singhal et al. 2015), as well as angiosperms, such as wheat (Saintenac et al. 2009), maize (Li et al. 2015), monkeyflower (Hellsten et al. 2013), and *A. thaliana* (Wijnker et al. 2013; Choi et al. 2013). In addition, the same pattern is observed in dogs (Canidae family), where PRDM9 was lost relatively recently (Axelsson et al. 2012; Auton et al. 2013). In these PRDM9-lacking organisms, chromatin structure is often invoked as an explanation of recombination hotspot conservation upstream of genes (Wu and Lichten 1994; Lichten 2008; Berchowitz et al. 2009). Nucleosome occupancy is depleted in regions where the DNA needs to be accessible, such as for the purposes of transcription. Promoter regions upstream of genes thus tend to display greater nucleosome depletion, which may in turn allow for recombination machinery to more easily induce double strand breaks in these regions (Pan et al. 2011; Lam and Keeney 2015). Our observations of elevated recombination rate immediately flanking genes suggest a similar mechanism acting in the *C. reinhardtii* genome and show that this trend is even more widespread, extending to green algae.

However, we observe that despite the trend above, recombination rate drops in shorter intergenic tracts. The *C. reinhardtii* genome is remarkably gene dense, with ∼17,700 intron-rich genes (Merchant et al. 2007), together spanning 85.8% of the genome. Over 90% of intergenic tracts are <2 kb, but the elevation of recombination rate near genes is primarily driven by the ends of longer intergenic tracts, whereas shorter tracts display far lower recombination rates despite being equally gene proximate. There are a few possible explanations for this trend: First, that shorter intergenic tracts may be the space between functionally related gene clusters, where suppressed recombination has evolved to maintain coadapted gene complexes (Pál and Hurst 2003; Poyatos and Hurst 2007); second, these gene complexes may share common regulatory sequence upstream of the clusters, such that short intergenic tracts between genes may not be in open chromatin and therefore are less susceptible to double-strand break formation; last, these short intergenic tracts may be dense with conserved functional sequence, and if recombination is mutagenic, historical crossovers may have been purged through purifying selection, reducing the effect of recombination in these tracts. Regardless of the underlying mechanism, in species where genes are tightly packed, there may not be sufficient intergenic space or suitable conditions to localize crossovers in the regions immediately flanking genes as is otherwise observed in other species lacking PRDM9.

We find a positive correlation between GC content and local recombination rate at fine scales. Our result is consistent with a trend seen in other organisms such as yeast (Gerton et al. 2000), mouse (Jensen-Seaman et al. 2004), and humans (Fullerton et al. 2001). There are several possible explanations for this trend: First, that GC-biased gene conversion is leading to increased GC substitutions at gene conversion tracts; second, that recombination preferentially initiates in GC-rich regions; third, that there is more efficient selection for GC content in regions with higher recombination rates (Kliman and Hey 1993; Campos et al. 2012). A recent study revealed weak effects of GC-biased gene conversion from the genome sequences of 27 *C. reinhardtii* tetrads, in concert with a low overall rate of gene conversion, thus indicating a minor role for biased gene conversion in the evolution of the *C. reinhardtii* genome (Liu et al. 2018). However, at the 2-kb scale, we obtain a stronger correlation than Liu et al., who report correlations in window sizes ranging from 10 to 200 kb, but we also do not obtain a significant correlation at broader (1 Mb) scales. A stronger GC-recombination correlation when considering historical recombination events suggests that the effects of weak forces governing fine-scale base composition may be more apparent over longer evolutionary timescales.

Using a crossover data set, we find that recombination correlates with nucleotide diversity across the genome of *C. reinhardtii*, indicating the action of selection at linked sites (fig. 3*B*). Theory predicts that the correlation of recombination and diversity arises as a consequence of background selection and/or selective sweeps reducing diversity in regions of low recombination (Charlesworth et al. 1993; Cutter and Payseur 2013; Campos et al. 2017). Our result suggests that selection on linked sites is a strong determinant of standing genetic variation in *C. reinhardtii*. Given that *C. reinhardtii* is likely to have a very high effective population size (Ness et al. 2012), it is expected that many mutations will be effectively selected (i.e., $N_{e}s>1$) (Kimura 1962). However, although the effective population size is very large, the relatively infrequent rate of sex (see below) means that the effective recombination rate is not particularly high relative to obligately sexual species. The interaction of a large *N*_e_ facilitating efficient selection alongside reduced recombination due to a facultatively sexual life cycle means that the influence of selection at linked sites may be pronounced in the genome and modulated less by recombination rate per se than would be the case in obligate outcrossers. This principle may be more widespread in unicellular eukaryotes, which live in large populations that are only periodically sexual.

Finally, by integrating lab- and population-based measures of recombination and mutation, we have estimated the rate of sex in *C. reinhardtii* to be one meiosis approximately every 840 asexual generations. The frequency is higher than estimates in two yeast species, *Saccharomyces cerevisiae* (*∼*50,000 generations, Ruderfer et al. 2006) and *S. paradoxus* (*∼*1,000–3,000 generations, Tsai et al. 2008), and is substantially more than the estimated rate of sex in *O. tauri* (*∼*94,000 generations, Blanc-Mathieu et al. 2017). However, the method we used to estimate the frequency of sex is subject to numerous assumptions, especially neutrality and demographic equilibrium; furthermore, if estimates of *ρ* are reduced by the effects of reduced diversity due to selection, it may downwardly bias our estimate of the frequency of sex, meaning that the true rate of sex in nature may be higher. Our estimate of sex occurring every 840 generations may point toward a seasonal ecology in *C. reinhardtii*. Although the precise rate of cell division in nature is unknown, lab cultures exhibit 2–3 doublings every 24 h (Bernstein 1964; Jones 1970; Harris et al. 2009) which means that 840 generations would take 336 days on average. Considering the fact that sex is induced when conditions worsen and zygotes are resistant to freezing, desiccation, and other environmental stressors (Morris et al. 1979; Suzuki and Johnson 2002; Harris et al. 2009), it is plausible that populations of *C. reinhardtii* in Quebec overwinter as zygotes, undergoing a sexual cycle approximately once per year.

Taken together, our results show that recombination in *C. reinhardtii* is punctuated, in terms of both the many recombination hotspots within the genome as well as the relative infrequency of sexual reproduction. Within-genome patterns in recombination rate patterns are consistent with other PRDM9-lacking organisms in localizing around genes. Interestingly, recombination in intergenic DNA is concentrated in the large intergenic regions between clusters of tightly packed genes. As with many microbial species, the effective population size of *C. reinhardtii* is very large, but this does not translate to a very high population recombination rate due to the fact that most generations are asexual. This leads to strong effects of selection at linked sites, which may be a scenario common in microbial eukaryotes more broadly. The periodic nature of recombination in microbial eukaryotes is likely a key difference in their population genetics which will need to be accounted for in future studies.

## Data Availability

Strains used in this study (supplementary table S1, Supplementary Material online) are available from the Chlamydomonas Resource Center (chlamycollection.org). Short read data are available at the European Nucleotide Archive under study accession ERP109393. All scripts used in this work are available at https://www.github.com/aays/reinhardtii-ld-rcmb (main analyses) and https://www.github.com/aays/ldhelmet-sims (validation of recombination estimation method via simulations). All statistical tests in this work were implemented using R 3.5.2 (R Core Team 2017).

## Supplementary Material

evaa057_Supplementary_Data
