# Supplementary material for: Recombination Rate Variation and Infrequent Sex Influence Genetic Diversity in Chlamydomonas reinhardtii
Source: Genome Biol Evol. 2020 Mar 17;12(4):370–80. doi: 10.1093/gbe/evaa057 (PMC7186780; doi:10.1093/gbe/evaa057)
Supplement: evaa057_Supplementary_Data [file evaa057_supplementary_data.pdf]

Supplementary Information

Strain	Collection Location/Year	Mating type
CC-2936	Montreal, Quebec/1993	+
CC-2937	Montreal, Quebec/1993	+
CC-3060	Farnham, Quebec/1995	+
CC-3064	Farnham, Quebec/1995	+
CC-3065	Farnham, Quebec/1995	+
CC-3068	Farnham, Quebec/1995	+
CC-3071	Farnham, Quebec/1995	+
CC-3076	Montreal, Quebec/1995	+
CC-3086	Montreal, Quebec/1995	+
CC-2935	Montreal, Quebec/1993	−
CC-2938	Montreal, Quebec/1993	−
CC-3059	Farnham, Quebec/1995	−
CC-3061	Farnham, Quebec/1995	−
CC-3062	Farnham, Quebec/1995	−
CC-3063	Farnham, Quebec/1995	−
CC-3073	Farnham, Quebec/1995	−
CC-3075	Montreal, Quebec/1995	−
CC-3079	Montreal, Quebec/1995	−
CC-3084	Montreal, Quebec/1995	−

Table S1: Field strains of *C. reinhardtii* used in this study. All strains were obtained from the *Chlamydomonas* Resource Center (`chlamycollection.org`).

Model 1: CDS versus intronic recombination
Response: Recombination rate (log10)

Predictor	β	t-value	p
SNP density (log10)	0.727	108.68	2.2×10^{-16}
annotation (in CDS: 1, intronic: 0)	0.309	21.66	2.2×10^{-16}
SNP density:annotation	0.058	5.96	2.39×10^{-9}

Table S2: Multiple regression of recombination rate by SNP density and annotation, contrasting recombination rate in coding exons versus introns.

Model 2: Long versus short intergenic tracts
Response: Recombination rate (log10)

Predictor	β	t-value	p
SNP density (log10)	0.550	31.19	2.2×10^{-16}
annotation (long: 1, short: 0)	0.240	9.69	2.2×10^{-16}

Table S3: Multiple regression of recombination rate by SNP density and annotation, contrasting recombination rate in long (>2 kbp) versus short (<2 kbp) intergenic tracts. Interaction term was nonsignificant and therefore excluded from the model.

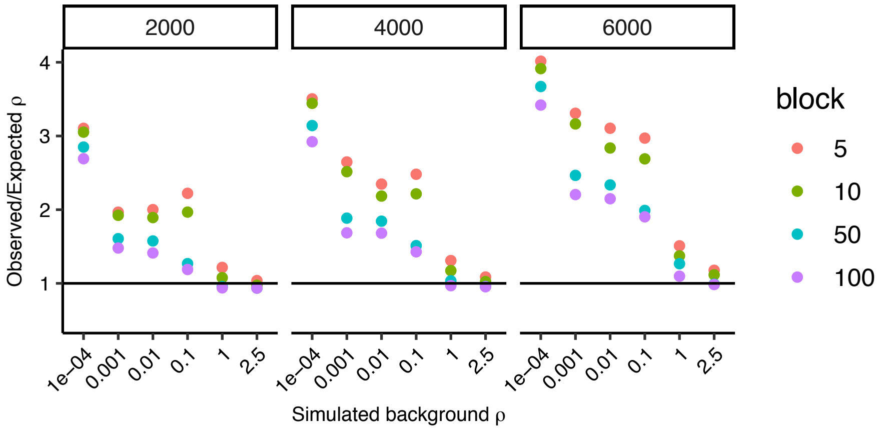

Figure S1: Observed/expected ρ across block penalties for simulated 1 Mb haplotypes generated under a range of background ρ values and varying hotspot sizes (2 kbp - 6 kbp).

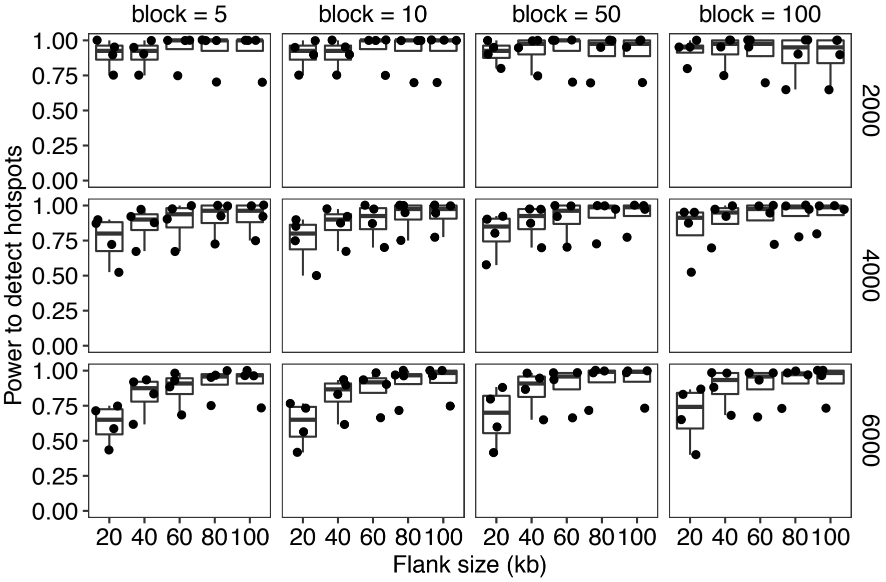

Figure S2: Power to detect hotspots under differing flank sizes and hotspot sizes around a central 2 kbp window. Hotspots were defined as 5-fold ρ elevations in a focal 2 kbp window over surrounding sequence. Power was defined as (1 - number of false negatives).

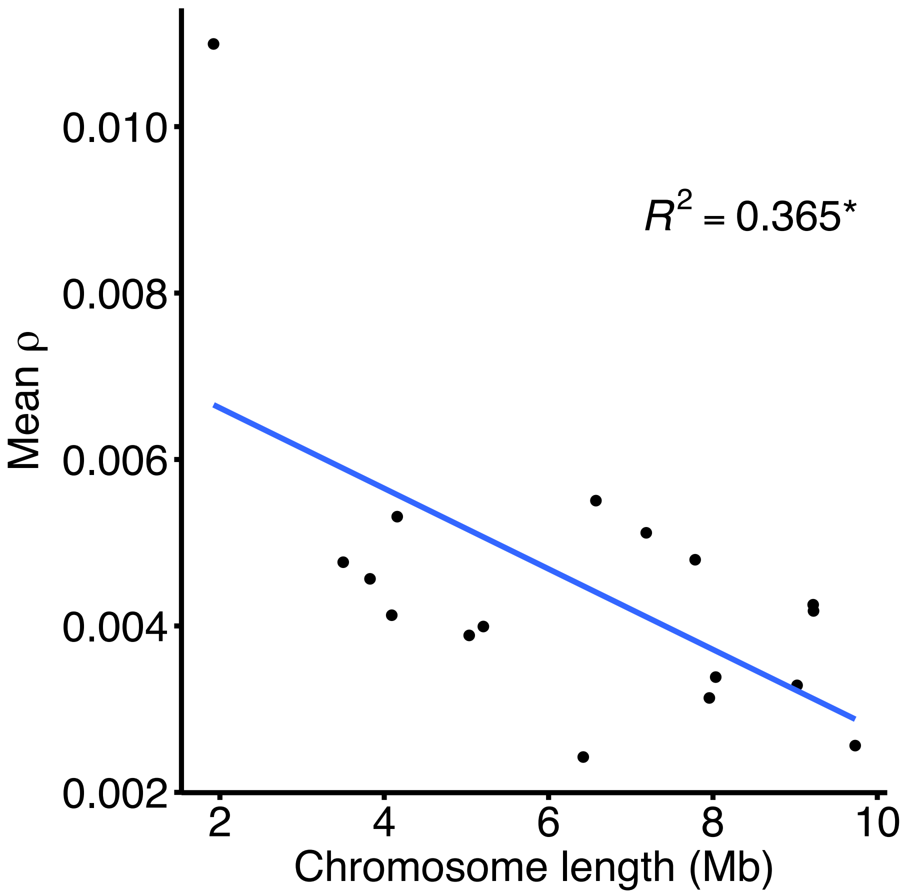

Figure S3: Chromosome length inversely scales with mean recombination rate ($R^2 = 0.4803$). Each point represents one of the 17 *C. reinhardtii* chromosomes.

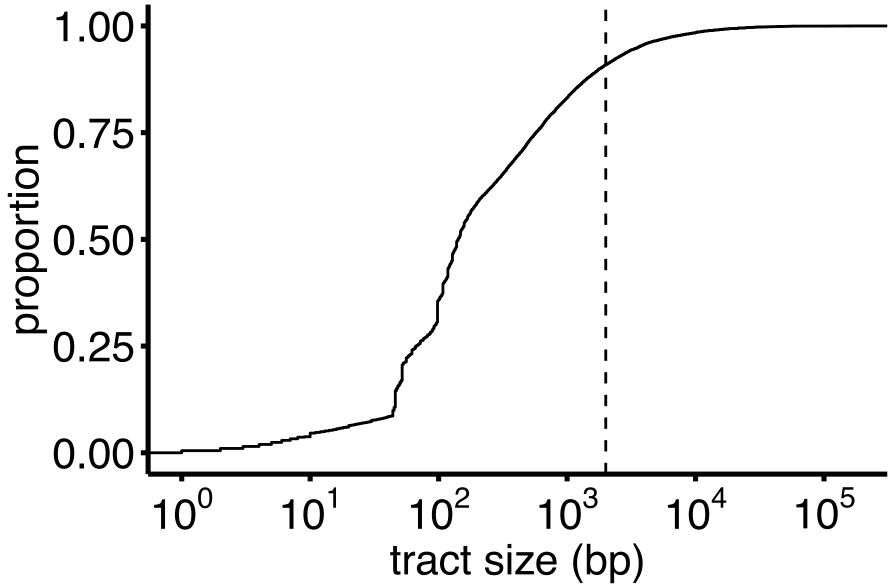

Figure S4: Cumulative frequency distribution of intergenic tract sizes, calculated using the *C. reinhardtii* v5.3 annotation. Dashed line indicates tract size = 2 kbp.
